# Nanoporous Titanium Oxynitride Nanotube Metamaterials with Deep Subwavelength Heat Dissipation for Perfect Solar Absorption

**DOI:** 10.1021/acsphotonics.3c00731

**Published:** 2023-09-08

**Authors:** Morteza Afshar, Andrea Schirato, Luca Mascaretti, S. M. Hossein Hejazi, Mahdi Shahrezaei, Giuseppe Della Valle, Paolo Fornasiero, Štěpán Kment, Alessandro Alabastri, Alberto Naldoni

**Affiliations:** † Czech Advanced Technology and Research Institute, Regional Centre of Advanced Technologies and Materials Department, Palacký University Olomouc, Šlechtitelů 27, Olomouc 78371, Czech Republic; ‡ Department of Physical Chemistry, Faculty of Science, Palacký University, 17. listopadu 1192/12, 779 00 Olomouc, Czech Republic; § Department of Physics, Politecnico di Milano, Piazza Leonardo da Vinci, 32, 20133 Milano, Italy; ∥ Istituto Italiano di Tecnologia, via Morego 30, 16163, Genoa, Italy; ⊥ Department of Electrical and Computer Engineering, Rice University, 6100 Main Street, Houston, Texas 77005, United States; # CEET, Nanotechnology Centre, VŠB-Technical University of Ostrava, 17 Listopadu 2172/15, Ostrava-Poruba 708 00, Czech Republic; ○ Istituto di Fotonica e Nanotecnologie - Consiglio Nazionale delle Ricerche, Piazza Leonardo da Vinci, 32, I-20133 Milano, Italy; □ Department of Chemical and Pharmaceutical Sciences, INSTM and ICCOM-CNR, University of Trieste, via L. Giorgieri 1, Trieste 34127, Italy; △ Department of Chemistry and NIS Centre, University of Turin, Turin 10125, Italy

**Keywords:** metamaterials, perfect absorbers, titanium oxynitride nanotubes, nanosized porosity, photothermal water evaporation

## Abstract

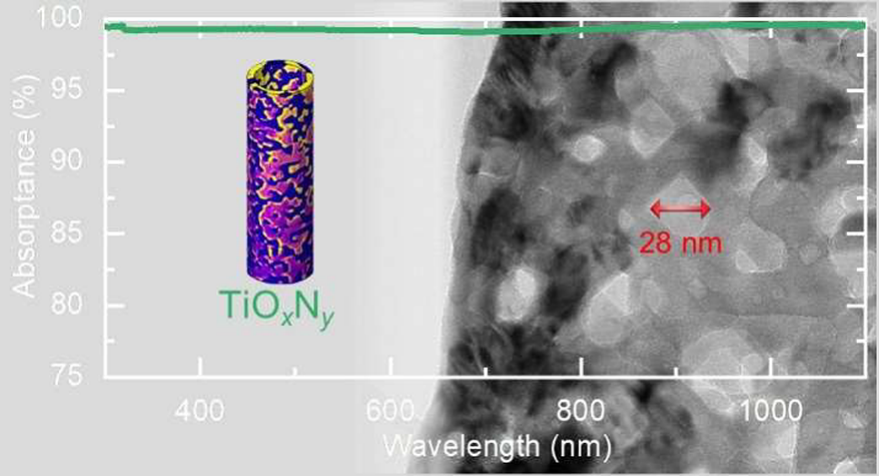

We report a quasi-unitary broadband absorption over the ultraviolet–visible–near-infrared range in spaced high aspect ratio, nanoporous titanium oxynitride nanotubes, an ideal platform for several photothermal applications. We explain such an efficient light–heat conversion in terms of localized field distribution and heat dissipation within the nanopores, whose sparsity can be controlled during fabrication. The extremely large heat dissipation could not be explained in terms of effective medium theories, which are typically used to describe small geometrical features associated with relatively large optical structures. A fabrication-process-inspired numerical model was developed to describe a realistic space-dependent electric permittivity distribution within the nanotubes. The resulting abrupt optical discontinuities favor electromagnetic dissipation in the deep sub-wavelength domains generated and can explain the large broadband absorption measured in samples with different porosities. The potential application of porous titanium oxynitride nanotubes as solar absorbers was explored by photothermal experiments under moderately concentrated white light (1–12 Suns). These findings suggest potential interest in realizing solar-thermal devices based on such simple and scalable metamaterials.

## Introduction

Simple and cost-effective broadband absorbers are highly desirable for developing large-scale solar-thermal technologies. Perfect light absorbers based on metamaterials have emerged as a promising solution to achieve satisfactory energy harvesting by efficiently absorbing the energy of incoming electromagnetic waves.^1,2^ Metamaterials are a class of engineered artificial materials typically consisting of arrays of subwavelength structures arranged in a periodic or nonperiodic manner.^3^ The interaction of the electromagnetic waves with the subwavelength structures of metamaterials, also called nanoresonators, determines their behavior and enables nanoscale light energy localization. By properly designing morphological parameters, the material, and the periodicity of the nanoresonators, it has been possible to reach desired permittivity (ε) and therefore to produce broadband absorbers.^4−6^ However, the main challenge in manufacturing metamaterials resides in the high cost, time-consuming, and intricate nature of the fabrication process.^7−9^ Hence, broadband absorbers must be developed based on Earth-abundant materials and industrially scalable preparation methods.

Broadband absorbers can be achieved effectively using plasmonic metamaterials because they exhibit excellent light confinement capabilities, originating from the plasmonic resonance they support.^6,10−13^ However, nanostructures made by the most conventional plasmonic materials (such as gold and silver) present limitations, such as low melting temperature, low chemical stability (in the case of silver), and high cost, which hinder their practical applications in plasmonic devices.^4,14,15^ To overcome these drawbacks, refractory plasmonic materials based on transition metal nitrides, particularly titanium nitride (TiN), have recently emerged as potential candidates for plasmonic applications.^14^ TiN has attracted significant attention due to its similar optical properties to gold, high thermal durability, chemical stability, mechanical hardness, adjustable permittivity, and good impedance matching to air within the visible range.^4,16−18^ Notably, TiN or titanium oxynitride (TiO_*x*_N_*y*_, *x* + *y* ≤ 1) in numerous morphologies, such as TiN nanotube (NT) arrays, cylindrical nanocavities, and hollow nanospheres, can be readily synthesized through cost-effective thermal nitridation process of titanium dioxide (TiO_2_) in the presence of ammonia (NH_3_).^19−24^ Moreover, Ti-based materials are abundantly available in the Earth’s crust, making them economical and sustainable candidates for large-scale fabrication.

Recent reports have confirmed that the 3D structure and morphology of TiO_2_ NT arrays with a high surface area can be effectively manipulated through the use of diverse electrolytes or anodization parameters, including voltage, time, and temperature,^25−28^ allowing a fine control on the optical properties of NTs.^29,30^ The distance between TiO_2_ NTs is a crucial factor in determining their photoelectrochemical performance, as spaced NTs featuring a higher air volume within the arrays exhibited greater light absorption compared to close-packed ones.^27,28,31^ However, these works focused on optimizing the optical response of spaced^27,28^ or close-packed^30^ TiO_2_ NTs for water-splitting applications. A detailed optical analysis of plasmonic TiO_*x*_N_*y*_ as a solar absorber material for photothermal applications is still missing.

In this work, titanium oxynitride nanotubes were experimentally and numerically studied as scalable broadband solar absorbers. The dimensional parameters and nanosized porosity were tuned by the synthesis process of electrochemical anodization and thermal nitridation in an ammonia atmosphere. The so-obtained nanotubes revealed exceptional broadband absorption capability, and numerical simulations showed that such nearly perfect absorption arise from the plasmonic character of the array and, crucially, from the presence of nanovoids in the nanotube walls introduced by the nitridation step. The fragmented electric permittivity associated with the nanotubes’ porosity induces intense hot spots and extremely localized electromagnetic dissipation within the porous regions, enabling large and broadband absorption. Such a heat dissipation mechanism cannot be captured through conventional effective medium theories, typically employed to reproduce large-scale optical effects associated with relatively small geometrical features.

To study such phenomena, we investigated a series of spaced TiO_*x*_N_*y*_ NTs with different lengths (0.6–3.2 μm), density, and degrees of nitridation as broadband solar absorbers. TiO_2_ NTs were prepared by electrochemical anodization and subsequently converted to TiO_*x*_N_*y*_ by thermal treatment under an NH_3_ atmosphere. Nanoporous TiO_*x*_N_*y*_ NT arrays demonstrated nearly unitary broadband absorption, i.e., >99%, in the 300–1100 nm range. Numerical simulations revealed that such an effect was related to light-trapping phenomena in the nanovoids in the tube walls. Fine control of the anodization parameters and nitridation temperature directly affected the NTs geometry (such as length and spacing) and oxide/nitride fraction, thus leading to optimal light absorption. As a result, the photothermal properties of the so-obtained TiO_*x*_N_*y*_ NTs were investigated by temperature measurements and solar steam generation experiments. In particular, under moderately concentrated light, the optimized nanotubes generated 235 °C and a solar-steam evaporation rate of 18 kg h^–1^ m^–2^. This work introduces the possibility of realizing cost-effective and scalable metamaterial absorbers based on titanium oxynitride that could be employed in large-scale or decentralized solar-thermal devices.

## Results and Discussion

The realization of NT-based broadband solar absorbers was first addressed by optimizing the tube lengths (1–6.8 μm) and diameter (85–245 nm) through anodization parameters. To this purpose, the effect of the applied voltage in the range of 20–60 V for 6 h was thoroughly studied, which led to highly spaced and aligned TiO_2_ NTs with a fairly regular arrangement (Figure S1). Such anodization parameters were chosen to ensure mechanical stability and a high morphological quality of the tube structure (Figure S2). The so-obtained TiO_2_ NTs exhibited a strong light absorption only for light energies >3.2 eV, i.e., higher than the electronic bandgap of TiO_2_ (Figure S3). In order to achieve perfect absorption, the NTs were thermally treated in air at 450 °C and then in NH_3_ at two different temperatures, i.e., 700 and 900 °C, which led to plasmonic TiO_*x*_N_*y*_ NT arrays. Hereinafter, NTs anodized at 20, 25, and 30 V and nitridated at 700 °C will be labeled as samples #1, #2, and #3, while those anodized at 60 V and nitridated at 700 and 900 °C will be referred to as samples #4 and #5, respectively.

**Figure 1:**
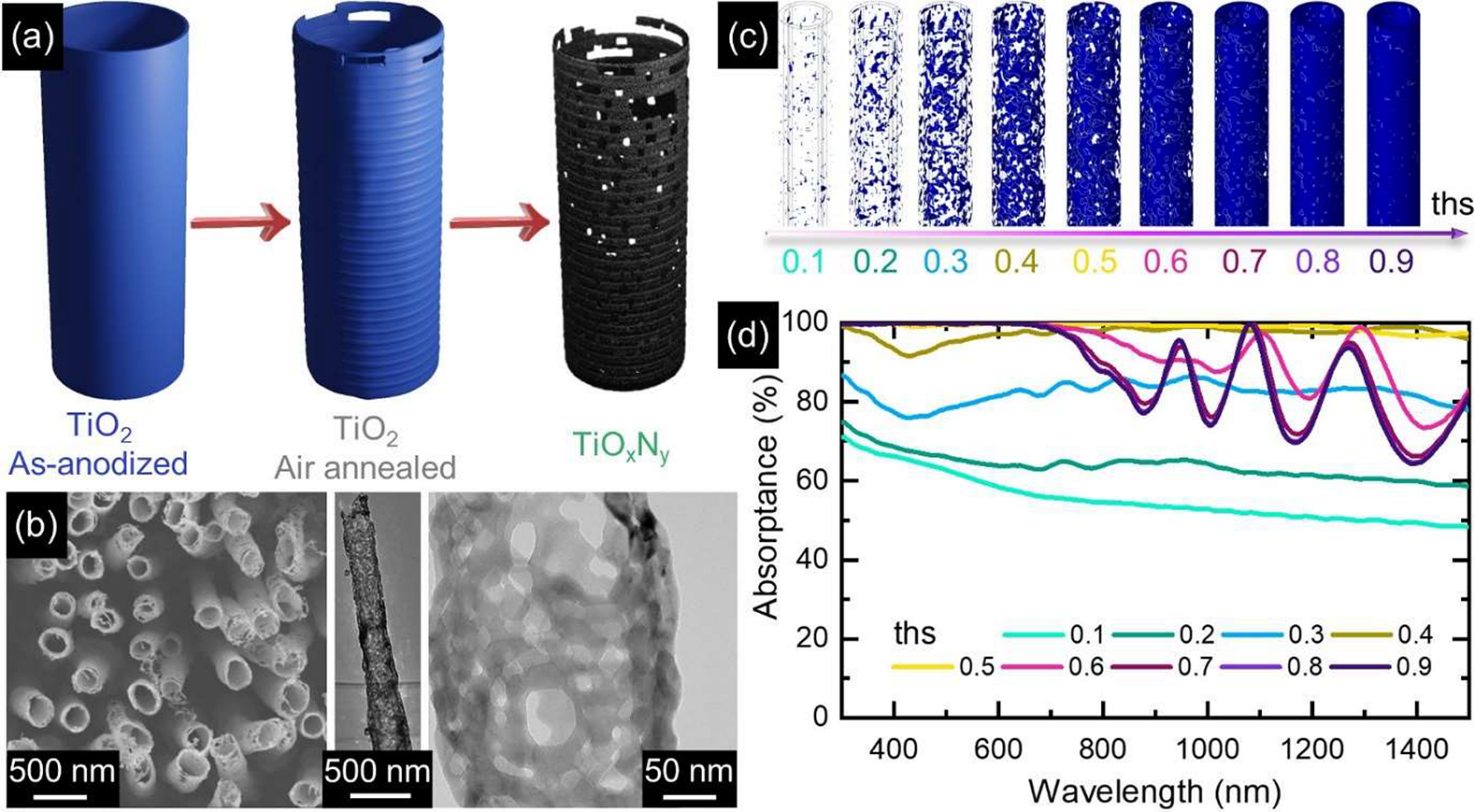
Nanoporous TiO_*x*_N_*y*_ NT arrays for broadband perfect absorption. (a) Schematic summary of morphological changes of NTs during conversion of TiO_2_ to TiO_*x*_N_*y*_. (b) SEM (left) and TEM (middle and right) images of NT arrays anodized at 60 V for 6 h and nitridated at 900 °C. (c) Sketch of the numerical model mimicking the NTs morphology, where the tube porosity is explicitly accounted for by creating a random space-dependent 3D map of permittivity, shown here, mixing voids and metal. The degree of porosity (i.e., metal content against voids) is controlled numerically by a threshold parameter (*ths*). (d) Simulated absorption spectra for an exemplary NT array on a Ti substrate by varying the threshold parameter *ths*, i.e., for increasing metal-to-void ratio. Numerical calculations considered the following geometrical parameters: outer diameter, 236 nm; wall thickness, 22.5 nm; length, 3.18 μm; array periodicity, 543 nm.

Figure 1a shows a schematic overview of the preparation of TiO_*x*_N_*y*_ NTs, while Methods and Supporting Information provide a detailed description of the fabrication process and properties of as-anodized and nitridated NTs. As a result of the nitridation process, the NTs morphology revealed a high degree of porosity characterized by nanovoids distributed throughout the tube walls (Figure 1b). The formation of porosity in the nitridation process is initiated by the partial decomposition of NH_3_ into N_2_ and H_2_ gases at an elevated temperature. Subsequently, oxygen atoms in TiO_2_ are extracted by hydrogen, resulting in oxygen vacancies that can be replaced by nitrogen. In addition, the imbalance of diffusion rates of ions, i.e., the Kirkendall effect, leads to the formation of NTs with a high degree of porosity.^32,33^ Since the size of these voids (between ∼3 and 40 nm) is significantly smaller than the wavelength of the incident light, they can play a crucial role in enhancing NTs absorption via plasmonic effects.^34^ On the other hand, the unique properties of nanoporous materials enable them to reduce the permittivity of a mixture compared to its original material and can further minimize the permittivity difference at the air-absorber interface. As a result, an antireflection effect can be obtained, making nanoporous materials close to a blackbody absorber.^34−37^

A numerical model was developed to better understand the role of NTs’ porosity on their light absorption. For an accurate description of the light-matter interactions within the system, electromagnetic simulations employed an original approach to account for the peculiar nanometric features across individual NTs. Specifically, when considering the materials’ optical properties, a wavelength- and space-dependent map of permittivity ε_NT_(λ,***r***) was associated with the NT numerical domain. Such a 3D map, defined as a combination of the permittivities of air (ε_Air_ = 1) and of the plasmonic material (TiN, taken from ref.^24^), was built numerically to exhibit spatial features over tens of nanometers, i.e., comparable to the typical size of the pores in the experimental structures. This strategy enabled *de facto* mimicking of the NT nanoporosity within the electromagnetic problem, by mixing voids and metal on a deep sub-wavelength scale across the tube wall. Moreover, the degree of porosity related to the metal-to-void volume ratio could be adjusted via a threshold parameter, *ths*. In formulas, by starting from a 3D normalized random pattern *c*(**r**) defined in each point **r** of the NT domain, the porous permittivity of the tube was assigned based on the value of *ths* as follows:
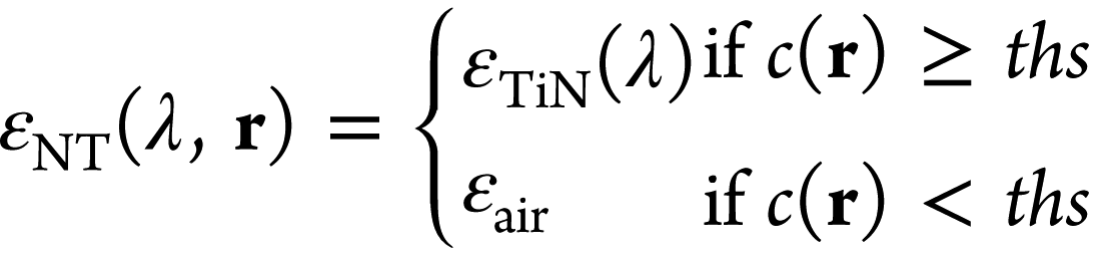
1with ε_air_ (ε_TiN_) the air (TiN) permittivity, so that setting *ths* = 0 provides a NT composed of air only, while *ths* = 1 corresponds to the case of a fully compact metallic structure. For intermediate values of *ths*, the corresponding metal-to-void volume ratio can be numerically computed based on the specific random function *c*(**r**) considered in the simulation and the NT total volume. Further details and technical aspects of implementing our approach are provided in Methods.

An exemplary set of porous permittivity maps ε_NT_, featuring different degrees of porosity as a function of the threshold value, is displayed in Figure 1c. Despite being associated with the same NT numerical domain, such distributions describe substantially different light–matter interaction scenarios. The extreme case of *ths* = 0.1 provides a NT mostly made of air with nanometric fragments of metal, whereas ε_NT_ with *ths* = 0.9 is essentially equal to ε_TiN_ everywhere in the NT (the metal-to-void volume fraction for the considered tube is computed to be larger than 99%). Intermediate values of *ths* are associated with permittivity spatial distributions mixing the metal content and air, with the former increasing compared with the latter for increasing thresholds. Importantly, the numerical results of our model indicate that varying ε_NT_, associated with different degrees of nanostructure porosity, dramatically impacts the optical response of the corresponding NT array. In particular, Figure 1d reports the absorption spectra computed over a broad spectral range (300–1500 nm) for a NT array with fixed geometrical parameters, where the porous permittivity of the individual tubes changes with *ths* according to the schematics depicted in Figure 1c. In these conditions, when the threshold is lower than 0.2, i.e., the NT is mainly made of air, a relatively flat absorption is obtained over the entire spectrum, ranging from 80% to values as low as 50%. On the other hand, when *ths* exceeds 0.7, and the metal content is much larger than the voids fraction, absorption is almost unitary at short wavelengths but abruptly drops by ∼20% close to 700 nm. At longer wavelengths, the numerical model predicts oscillations in absorption, to be possibly ascribed to a combination of longitudinal modes supported by the single NTs, featuring a pronounced aspect ratio (an outer diameter of 236 nm and a length of 3.18 μm are set in the simulations) and coupling modes between neighbors in the array. The most intriguing results, however, are obtained for thresholds close to 0.5 (corresponding to metal-to-void volume ratios approximately equal to 50%, depending on the specific simulated system). For those values, the computed absorption approaches unity over the entire spectral range under consideration. This observation indicates a non-negligible mixing of metal and air at the nanoscale as the key element to promote an almost unitary absorption system over a broad bandwidth. Additionally, the existence of an optimum for *ths* suggests that tailoring the degree of porosity across the NTs is pivotal in engineering similar perfect solar absorbers.

**Figure 2:**
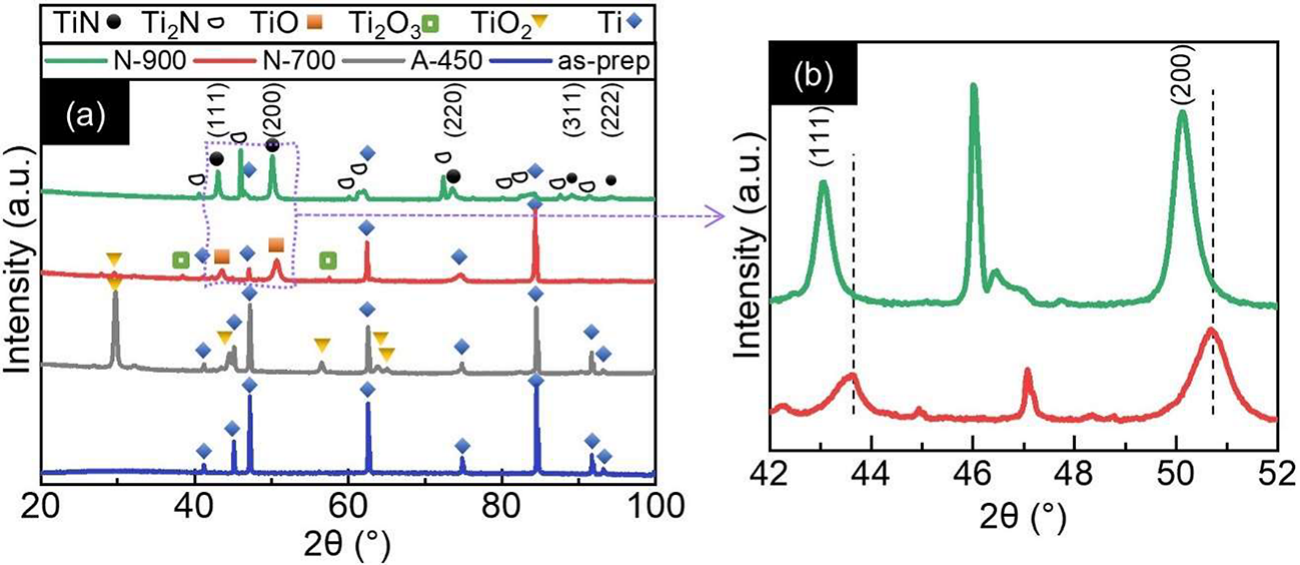
Impact of thermal treatment on chemical composition of NTs. (a) X-ray diffraction patterns of (blue) as-prepared and (gray) air-annealed TiO_2_ nanotube arrays and nitridated samples at different temperatures of (red) 700 and (green) 900 °C. (b) A magnified section of the patterns that emphasizes the shift of the (111) and (200) crystallographic orientations by increasing the nitridation temperature.

Apart from nanosized porosity resulting from the nitridation process and the significant impact of porosity on the light absorption behavior, various morphological and chemical characteristics were modified through the nitridation. The crystal structure of the as-prepared, annealed, and nitridated nanotubes was investigated by X-ray diffraction (XRD, Figure 2a). The XRD pattern of the as-prepared TiO_2_ nanotubes exhibited only the characteristic peak of metallic Ti originating from the substrate, thus confirming the amorphous nature of the as-anodized samples. Upon annealing at 450 °C, the nanotubes crystallized to the anatase phase with a limited rutile fraction (evidenced by a small peak at 2θ ≈ 32°). Subsequent nitridation at 700 °C promoted a partial phase transformation to TiO_*x*_N_*y*_, as suggested by the nearly fading peaks of TiO_2_ and Ti_2_O_3_ together with the onset of the pattern of the cubic Ti(O,N) structure (space group *Fm*3̅*m*). By increasing the nitridation temperature to 900 °C, the peaks associated with the oxide phases (TiO_2_ and Ti_2_O_3_) disappeared. Meanwhile, the peaks associated with the Ti(O,N) structure increased in intensity and shifted to lower diffraction angles and additional reflections appeared, which could be associated with the tetragonal Ti_2_N phase. This is further illustrated in Figure 2b, which clearly shows the shift for the (111) and (200) peaks with the nitridation temperature. Such an observation was further confirmed by retrieving the lattice parameter of the nitridated samples using Bragg’s law for the cubic crystal systems (Table S1). In particular, the average lattice parameter increased from ∼4.18 to ∼4.23 Å for the samples nitridated at 700 and 900 °C, respectively (see the Supporting Information for further details). These results, therefore, suggest the increase of nitrogen content with the annealing temperature. For the sake of simplicity and because a residual degree of oxidation could not be ruled out, the nitridated nanotubes are termed TiO_*x*_N_*y*_, regardless of the nitridation temperature.

**Figure 3:**
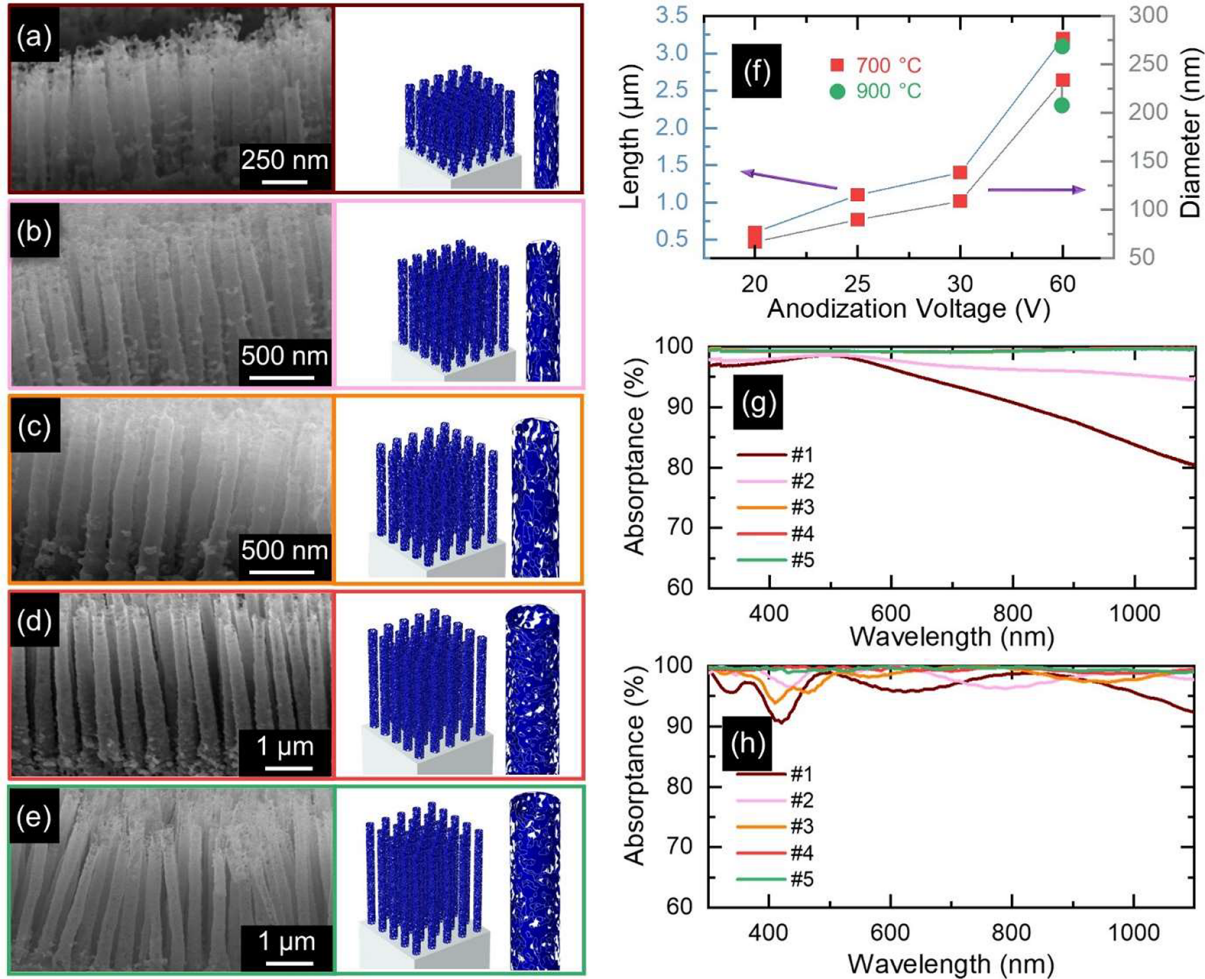
Morphology and absorption spectra of NT arrays. (a–e) Cross-sectional SEM images and sketches of the geometries (the geometrical parameters considered in the simulations are set based on SEM analysis of the samples) simulated in the numerical model mimicking the experimental TiO_*x*_N_*y*_ NT arrays (threshold value set to 0.5 in calculations) anodized for 6 h at (a) 20, (b) 25, (c) 30, and (d, e) 60 V and nitridated at (a–d) 700 and (e) 900 °C. (f) Effects of the applied potential and nitridation temperature on the average diameter (right-hand side axis) and length (left-hand side axis) of NT arrays. (g, h) Experimental and calculated optical absorption of NTs, respectively.

The combination of chemical and morphological alterations can significantly impact the optical properties of NT arrays (Figure 3). In particular, upon nitridation at 700 °C, the overall morphology of NTs was preserved (Figures 3a–d and S4a–c), while a shrinkage in diameter and length was observed because of a sintering effect (Figure 3f) in contrast to that of their as-anodized counterparts (Figure S1j). For instance, the average length and diameter of the as-anodized NTs at 60 V decreased from 6.8 μm and 245 nm to 3.3 μm and 234 nm, respectively, after nitridation at this temperature. Moreover, TEM images revealed a roughening effect at the NT wall with numerous nanopores at the upper part of the NTs wall, which can be attributed to diffusion kinetics of the O and N atoms in the nitridation treatment, while the same did not occur at the lower part of the NT wall (Figure S4c).

On the other hand, nitridation at 900 °C did not affect the overall NTs morphology, but produced a slight bend of the tubes toward each other (Figures 1b and 3e). In addition, the degree of reduction in the average diameter of NTs was higher compared to those nitridated at 700 °C, and it shrank from 245 nm in as-anodized NTs to 207 nm in the sample nitridated at 900 °C (Figure 3f). The average length of NTs experienced a slight reduction in comparison with samples nitridated at 700 °C. Also, the NTs’ surface became entirely porous wherein the upper region demonstrated a greater level of porosity in comparison to the lower region (Figure 1b) and with bigger pores than nitridation at 700 °C (Figure S4c).

The experimental optical characterization (Figure 3g) of samples #5, #4, and #3 demonstrates broadband and almost unitary absorption in the wavelength region 300–1100 nm. Such a broad absorption bandwidth can be attributed to various factors, including NTs porosity, length and diameter (density) of NTs, and the length of the light pathway inside the NTs. The NT walls exhibit a gradual decrease in porosity (Figure S4c) and a concurrent increase in thickness (e.g., sample #4, with a thickness range of 30 to 110 nm) from top to bottom. This morphological variation results in a slight increase in the permittivity of NT arrays from the upper to lower portions and a further antireflection effect.^38,39^ In addition, after light passes through the air-absorber interface without reflection, nanovoids in the NT arrays allow for significant plasmonic field confinement in deep subwavelength volumes, promoting efficient electromagnetic dissipation.

Absorption is also noticeably influenced by the length and diameter (i.e., density) of the NTs. Generally, in addition to porosity, the fact that (i) NTs exhibit different lengths and especially diameters (hence, changing the center-to-center distances) in each specific sample (e.g., Figure 1b); and (ii) periodicity is broken on a large scale, i.e., NTs are randomly arranged within the array, leads to the scrambling of resonances and consequently the occurrence of broadband absorption spectra.^40−42^ Specifically, shorter NTs (e.g., samples #1 and #2) displayed high absorption properties at shorter wavelengths, whereas longer NTs extended absorption effectively at longer wavelengths. Longer NTs exhibited significantly lower reflectance and a flatter spectral profile over the entire spectral range than their shorter counterparts, making them a promising candidate for achieving near-unity absorption across the whole solar emission range of wavelengths. This is because the domain extension among the NTs is an additional factor to enhance absorption, which increases with increasing length of NTs. In other words, the multiple scattering of light between and inside of NTs and across the nanosized porous NT network, which significantly prolongs the optical path and increases the possibility of light absorption, leads to ultrahigh absorption.^10,12,38^ While in shorter NTs, light energy can be reflected from the interface between the NTs and substrate because of a lack of sufficient light pathway and inadequate light dissipation.

Additionally, it was observed that the absorption of a sample decreased as the diameter of NTs reduced, and the sample with the shortest diameter or highest density of NTs, #1, demonstrated the lowest absorption. This effect can be attributed to the lower air density through the regions between NTs and the higher permittivity difference at the interface of NT arrays and air. The broadband absorption resulting from the combination of these factors gives an even higher absorption compared to previous reports on TiN nanoarrays.^4,43,44^ For example, a 94% absorption was attained in ref (45) by coating TiN on Si nanopillars and optimizing the effect of geometrical parameters of the nanostructure arrays.

To support the experimental observations, the model previously introduced was employed to compute the optical response of the different samples, and the average geometrical parameters estimated from the SEM analysis were first used to build the numerical NT domain for each of them.

The approach outlined before to create porous optical properties across the NT was then applied to model the electromagnetic interaction with the nanostructures sketched in Figure 3a–e (right panels), which show the considered single porous NT and the corresponding array for the five samples. The computed absorption spectra for all of the samples, reported in Figure 3h, reasonably agree with the experimental findings (Figure 3g). Almost perfectly flat absorption is obtained over the whole spectrum for the five nanostructure arrays. The trend with varying NT dimensions is also qualitatively retrieved by the numerical results, capturing the decrease in absorption at longer wavelengths for sample #1. The observed mismatch with experiments (in particular, the oscillations across the simulated spectra, absent in the measured ones) is expected to be due to the fact that models consider an infinite, perfectly periodic array of precisely identical NTs, thus neglecting the size dispersion and random spatial arrangement of the nanostructures within the experimental arrays.

**Figure 4:**
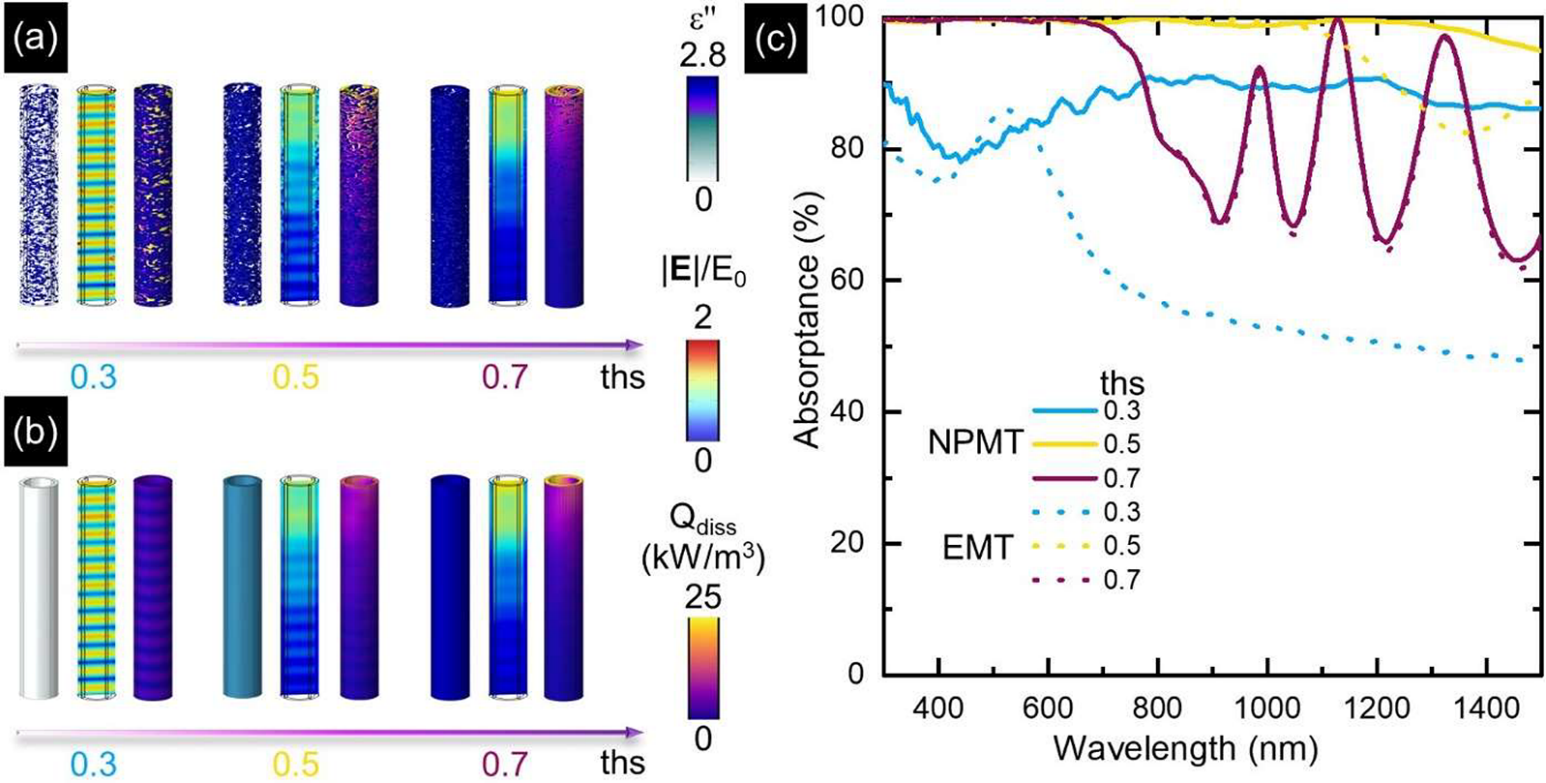
Modeling porosity across NTs: failure of the effective medium theory. (a) From left to right, a 3D map of a single NT permittivity (imaginary part, ε′′), the spatial distribution of electric field enhancement factor (|*E*|/*E*_0_), and dissipation power density (*Q*_diss_) calculated at a representative wavelength of 532 nm by varying the threshold parameter from 0.3 (left), 0.5 (middle), to 0.7 (right). Numerical results refer to sample #4 (outer diameter 293 nm, wall thickness 30.5 nm, length 3.3 μm, array periodicity 586 nm). (b) Same as (a) when effective medium theory (EMT) is employed in the simulations to define an effective uniform permittivity across the individual NT, mixing air and TiN permittivities. EMT-based calculations considered the metal-to-void ratio set by the threshold value used in the corresponding simulations in (a). (c) Numerically computed absorption spectra for the exemplary NT array considered (geometrical parameters for sample #4) for varying values of the threshold *ths*, by applying the original modeling approach here proposed (NPMT, solid curves) and EMT (Bruggeman formalism, dotted curves), respectively.

Furthermore, our model, termed nanoporous medium theory (NPMT), ascertained the origin of such a high broadband absorption, rationalized in terms of the nanometric discontinuities introduced by porosity in the tube permittivity. To show that, the results of our simulations were compared with the outcome of similar calculations using effective medium theory (EMT) to account for nanoporosity across the tube. Specifically, the electromagnetic problem was solved by pursuing either our NPMT modeling approach or an EMT-based one (implementing Bruggeman formulas,^46^ see Methods) for the geometrical parameters of sample #4 and values of the threshold equal to 0.3, 0.5, and 0.7 as illustrative cases. Figure 4 summarizes the main results of such a comparison for an exemplary wavelength of 532 nm. Specifically, for each value of *ths*, Figure 4a,b displays, from left to right, (i) the permittivity (imaginary part) ε′′, (ii) the electric field enhancement |*E*|/*E*_0_, and (iii) the dissipated power density *Q*_diss_ across an individual NT. While NPMT (Figure 4a) explicitly accounts for the NT porosity through the nanometric features of ε_NT_, the EMT-based description (Figure 4b) assigns to the NT an effective permittivity, neglecting any nanoscale discontinuity. This results in a homogeneous permittivity (see the left insets for each threshold in Figure 4b), weighted over the relative content of the plasmonic material. Contrary to EMT, the description proposed in Figure 4a introduces pronounced and highly localized field enhancements over spatial scales much shorter than the geometrical size of the NT (compare the middle insets for each threshold in Figure 4a,b). Such localized features induce a scrambling effect of the tube resonant mode patterns and increase the electromagnetic dissipation in deeper regions along the height of the nanostructure itself (compare the right insets for each threshold in Figure 4a,b).

Besides discrepancies in the NT local optical properties, the two modeling approaches also predict substantially different global nanostructure responses. In particular, Figure 4c reports the simulated absorption spectra for the three threshold values of porosity considered (*ths* = 0.3, 0.5, and 0.7, corresponding to metal-to-void volume ratios of ∼4.7%, 51%, and 95%) when either NPMT (solid lines) or EMT (dotted) is employed. The most pronounced differences between the two models’ results are observed for the lowest degree of porosity (*ths* = 0.3, light blue curves). Our approach predicts a relatively flat (besides a slight dip around 450 nm) spectrum and values for absorption larger than 80% over the entire bandwidth. Conversely, EMT provides a spectrum featuring a peak at ∼500 nm, abruptly decaying around ∼600 nm and reaching values as low as 50% at longer wavelengths. According to the effective description of Bruggeman formulas, such an optical response is that of an array of NTs with homogeneous metal-like permittivity, the plasmonic character of which is rather poor (ε′ is small), given the large fraction of air present. On the contrary, when the porous system is predominantly made of metal (i.e., for *ths* = 0.7 when the air content in the porous NT is <5%, purple lines), the two modeling approaches lead to almost identical results. Given the small number of voids in the permittivity map, our porous NT is essentially equivalent to a compact tube with optical properties close to those of pristine TiN (compare ε′′ in the left insets for *ths* = 0.7 in Figure 4a,b). As such, the obtained spectra exhibit identical features: an almost unitary absorption until ∼600 nm and resonant modes providing oscillations excited for longer wavelengths (Figure 1d). Finally, for the intermediate value of *ths* = 0.5 (yellow curves), although the two simulations provide a similar high absorption until ∼1000 nm, the EMT-based spectrum drops at longer wavelengths, decreases to 80% and oscillates. The same does not occur when NPMT is used, leading to a quasi-unitary absorption up to 1500 nm. Based on this comparison, performed for the optimal threshold value used in Figure 3, we conclude that a porous ε_NT_ with nanoscale discontinuities is required to reproduce the broadband quasi-perfect absorption observed experimentally.

**Figure 5:**
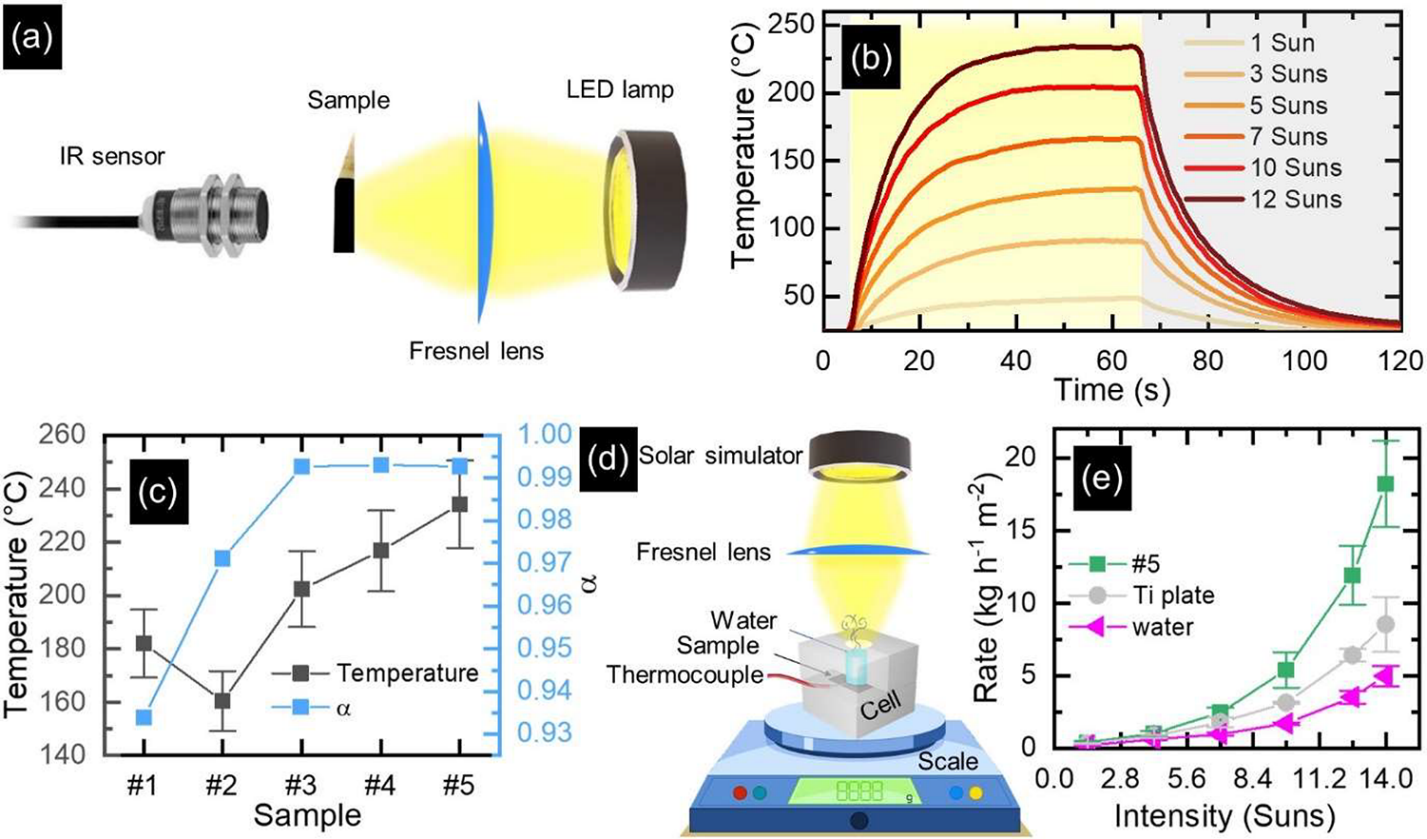
Photothermal applications of NT arrays. (a) The schematic setup for temperature measurement via an IR sensor. (b) Heating–cooling cycles of #5 NT arrays, anodized at 60 V for 6 h and nitridated at 900 °C, under different irradiation intensities of an LED lamp. (c) (gray, left-hand side axis) Maximum temperature (*T*_max_) extracted from the heating and cooling curves of different samples under 12 Suns irradiation of an LED lamp in comparison with the averaged solar absorptance of NTs (blue, right-hand side axis). (d) The schematic diagram of the steam generation setup. (e) Comparison of solar steam generation performance among sample #5, Ti plate, and water.

The exceptional absorption properties of TiO_*x*_N_*y*_ NTs make them ideal for photothermal applications. In this context, the photothermal behavior of NT arrays was investigated (Figure 5) by employing an infrared (IR) sensor owing to its noncontact nature and high accuracy.^47^ For this purpose, the heat generation of each sample was determined by placing the IR sensor on the backside of the sample while it was being illuminated with white light from an LED source (emission spectrum reported in Figure S5a) at different intensities, i.e., 1–12 Suns, where 1 Sun = 100 mW cm^–2^ (Figure 5a). By measuring the temperature of the samples over a period of time (2 min) by turning on and off the LED lamp, heating and cooling curves were generated. An example of heating and cooling curves is shown for sample #5 in Figure 5b. The sample temperature rose within ∼15 s, reached a steady temperature after ∼60 s, and quickly decreased when the light was switched off. As expected, the temperature measured under irradiation increased with an increase in the incident light intensity. The maximum temperature (*T*_max_) recorded for each sample was extracted from the corresponding heating–cooling curve at different irradiation intensities after 60 s of irradiation (Figure S5b). Figure 5c displays the results of this analysis under illumination at 12 Suns in comparison to the spectrally averaged solar absorptance (α). The α is a fundamental parameter in determining the fraction of incident solar radiation that is absorbed by NT arrays and can be calculated by using the following equation:
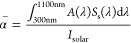
2in which *A*(λ) is the absorptance, and the spectral solar irradiance (AM 1.5G) is represented by *S*_s_(λ), the total irradiance by *I*_solar_, and the integration is performed over the experimentally measured wavelength range, i.e., 300–1100 nm. The results of this calculation (Figure 5c) indicate that all five samples demonstrated significantly high levels of solar absorption with values of α as high as 0.96. The highest value of α, greater than 0.99, was achieved in the case of NTs anodized at 30 and 60 V (samples #3, #4, and #5), and the value of this parameter gradually reduced as the anodization voltage decreased, reaching a minimum of 0.96 for sample #1, featuring NTs with the shortest length. On the other hand, these results also demonstrate that sample #5 generated the highest temperature compared to the other samples. This is likely due to its chemical composition and high absorption. Moreover, longer TiO_*x*_N_*y*_ NTs showed a higher temperature increase than their shorter counterparts, which can be ascribed to their higher surface area and higher light absorption.

One of the most promising applications of photothermal materials is the solar steam generation, which enhances the evaporation of water that occurs naturally under solar light by employing a photothermal material.^48,49^ The so-obtained water vapor can be collected into clean water, thus ensuring a simple approach to water desalination, purification, or distillation, even in remote locations. The solar steam generation performance of the samples was investigated by employing a custom setup (Figure 5d).^17^ Briefly, in each experiment, the sample was enclosed within a polytetrafluoroethylene (PTFE) cell together with 800 μL of water and a thermocouple, and the PTFE cell was placed on a high-precision balance and illuminated under solar-simulated light at different intensities (1.4–14 Suns) for 25 min to monitor the mass change of water (see Methods for additional details). In the absence of illumination, no measurable water evaporation occurred, while at high irradiation conditions, a substantial steam flow was continuously observed at the water surface (see Figure S6), except for water-only conditions. The presence of TiO_*x*_N_*y*_ NTs at the bottom of the water volume accelerated the kinetics of its evaporation, as also occurred by increasing the light intensity for a specific experimental condition, as expected (Figure S7). Such an observation clearly confirmed the photothermal effect of TiO_*x*_N_*y*_ NTs.

The performance in solar steam generation experiments can be evaluated from weight change data by computing the evaporation rate *ṁ* (kg m^–2^ h^–1^) based on this equation:
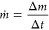
3where Δ*m* refers to the mass change of water during a specific time interval Δ*t*. Figure 5e presents an overview of the recorded evaporation rates for sample #5 and Ti foil, as well as for pure water. Sample #5 was chosen as a representative case that exhibited the highest temperature among all investigated nanotubes (Figure 5c). The water evaporation rate for all of the other samples was, indeed, slightly lower than that of sample #5 (Figure S8). The results (Figure 5e) indicate a negligible enhancement of the water evaporation rate for all samples at lower irradiation intensities, while at higher intensities the evaporation rate increased with a nonlinear trend. Such a nonlinear increase may be rationalized in terms of the exponential growth of light penetration through the bulk water on top of the NTs, and consequently higher conversion of light to heat and to the exponential increment of water vapor saturation pressure with temperature.^50^ Therefore, a synergy of these effects could increase the kinetics of water evaporation. In particular, the lowest evaporation rate was found for the water-only conditions (4.97 kg m^–2^ h^–1^ at 14 Suns), while the maximum value (18.20 kg m^–2^ h^–1^ at 14 Suns) was observed for sample #5, i.e., 3.66 and 2.13 times higher than that of pure water and Ti foil, respectively. In addition, it is also higher than the evaporation rate obtained in our previous study on TiN nanocavity arrays (∼15 kg m^–2^ h^–1^).^17^ Overall, the results obtained from different solar-thermal experiments, presented in Figure 5, suggest the possibility of employing TiO_*x*_N_*y*_ NTs as scalable photothermal materials in various technologies.

## Conclusions

In summary, TiO_*x*_N_*y*_ NT arrays were experimentally and numerically investigated as promising broadband solar absorbers. The dimensional parameters of the NTs were explored by tuning the conditions of electrochemical anodization and post-synthesis thermal treatments in NH_3_ atmosphere. Nearly unitary absorption over a wide wavelength range (300–1100 nm) was achieved by a combination of morphological features of the tubes, i.e., optimized length/diameter and nanosized porosity, and the plasmonic nature of TiO_*x*_N_*y*_. An original modeling approach was developed to rationalize such a high and broadband absorption, enabling us to accurately mimic the porous features of NTs at the nanometric scale. By numerically creating a map of permittivity mixing voids and metal across the individual NTs of the array, high and localized field enhancements were predicted on spatial scales much shorter than the geometrical size of the tubes, scrambling resonances, and promoting broadband electromagnetic absorption.

Contrary to the conventional description of porous media based on effective medium theories, our simulations reproduced the experimental observations, suggesting new insights into the light–matter interactions in porous nanostructures. Moreover, solar-thermal experiments demonstrated that longer TiO_*x*_N_*y*_ NTs generated higher temperatures and more steam under different irradiation intensities, ascertaining their potential as efficient photothermal materials. These findings propose the development of a simple and cost-effective metamaterial with near-unity absorption and demonstrate the significance of TiO_*x*_N_*y*_ NTs as promising candidates for various technological applications.

## Methods

### Sample Preparation

Spaced TiO_2_ NTs were prepared by electrochemical anodization in a two-electrode electrochemical cell, where a Pt sheet served as the counter electrode and a Ti foil (0.25 mm thick) was used as the working electrode. First, a commercial Ti foil was ultrasonically cleaned and degreased in acetone, ethanol, and distilled water for about 15 min successively and then dried under a nitrogen flow. Second, the foil was anodized in diethylene glycol (DEG) electrolyte consisting of 0.5 wt % NH_4_HF_2_ and 3.6 wt % H_2_O at different direct current (DC) voltages of 20, 25, and 30 V for 6 h at 50 °C as well as 60 V for 6 h at 40 °C. After anodization, the sample was completely washed with ethanol and water to remove the residual electrolyte and then dried in the nitrogen stream. Later on, in order to prepare crystalline TiO_2_ NTs, the NTs were annealed in air at 450 °C for 2 h by heating and cooling ramp of 2 °C/min. Subsequently, the NTs were also annealed under NH_3_ flow (10 mL/min) for 1 h at 700 and 900 °C (heating and cooling ramp of 5 °C/min) to obtain TiO_*x*_N_*y*_ NTs, and the obtained samples are denoted as #1, #2, and #3 to refer to samples that underwent anodization at 20, 25, and 30 V and nitridation at 700 °C, while labels #4 and #5 were used to refer samples anodized at 60 V and nitridated at 700 and 900 °C, respectively.

### Characterization

The morphology of the samples was characterized by scanning electron microscopy (SEM, Hitachi FE-SEM 4800) and transmission electron microscopy (TEM JEOL 2010). The crystalline structure was investigated by an X-ray diffractometer (XRD, PANalytical, Almelo, The Netherlands) with Co–Kα radiation (λ = 0.179 nm) operated in a Bragg–Brentano geometry. The optical properties were investigated by reflectance measurements with a Specord250 Plus spectrometer equipped with an integrating sphere (Analytik Jena GmbH, Germany) in the 300–1100 nm range and with a vacuum Fourier-transform infrared (FTIR) Vertex 80v spectrometer in the 1330–25000 nm range. The absorptance (*A*) was retrieved by reflectance (*R*) spectra as *A* = 1 – *R* by neglecting the transmittance (*T*) due to the opaque nature of Ti substrates in all the investigated spectral ranges.

### Temperature Measurement with IR Sensor

In order to accurately measure the temperature of the samples under irradiation, Fourier-transform infrared (FTIR) spectroscopy (Vertex 80v spectrometer, Bruker) was used to measure the samples back-surface reflectance at room temperature, which was then converted to emissivity by Kirchhoff law and averaged over the wavelength range corresponding to the IR sensor sensitivity range (8–14 μm; see Table S2). The samples were illuminated from the front side with a LED lamp (see the emission spectrum in Figure S5a), while an IR sensor (Omega OS-MINI802-D-C4) was placed at a 10 cm distance from the backside of the samples, thus allowing a noncontact measurement of the temperature. The samples were irradiated under different intensities (1–12 Suns, 1 Sun = 100 mW cm^–2^) by focusing the LED light with two lenses (i.e., a fused silica plano-convex lens, Thorlabs LA4984, and a Fresnel lens, Thorlabs FRP251). The light intensity was measured by a thermopile detector (Standa 11UP19K-30 H-H5) before each experiment.

### Water Evaporation Experiments

The solar-to-heat performance was evaluated by water evaporation experiments under solar-simulated light. The samples were placed within a thermally insulating polytetrafluoroethylene (PTFE) cell made of two parts to firmly hold the Ti substrate on which the NTs were grown. The top part of the cell had a cylindrical through-hole with a 1 cm diameter acting as a water reservoir and allowing illumination of the sample surface. A type K thermocouple (RS PRO, 0.075 mm diameter) was positioned on the top surface of the sample and the cell was placed on a high-precision electronic scale (Kern and Sohn GmbH, 0.1 mg accuracy). Afterward, 800 μL (800 mg) of deionized water was poured into the reservoir, and the cell was illuminated with a 1000 W solar simulator (Sciencetech A4 Lightline C250) equipped with an AM 1.5G filter and a Fresnel lens to focus the light to a circular spot, which matched the hole diameter of the top part of the PTFE cell (1 cm). Prior to experiments, the light power was measured with a thermopile detector (Standa 11UP19K-30 H-H5). Each water evaporation test for NT samples and pure water (without NT sample and as a comparison) was carried out with three replications by monitoring weight change during 25 min under different light illumination intensities from 1.4 to 14 Suns (1 Sun = 100 mW cm^–2^).

### Numerical Modeling

The numerical results presented in the main text have been obtained by employing a Finite Element Method (FEM)-based commercial software (COMSOL Multiphysics 6.0) to develop a three-dimensional (3D) model of the porous NTs under investigation. Full-vectorial electromagnetic simulations have been performed to determine the optical response of each system, treated as an infinite, perfectly periodic array of NTs lying on a Ti substrate and embedded in air. Maxwell’s equations are solved across the array unit cell, the geometrical parameters of which (that is, the NT length, external radius, and wall thickness along with the center-to-center distance between neighbors, assuming a squared lattice) were estimated from SEM analysis of the five experimental samples. A monochromatic linearly polarized plane wave at normal incidence has been considered, and ports formalism in the frequency domain has been employed to compute the electromagnetic behavior of the NT array in the spectral range between 300 and 1100 nm. Floquet periodic boundary conditions (BCs) were set at the lateral sides of the unit cell to simulate an infinite array, and perfectly matched layers (PMLs, with scattering BCs beyond such domains) were defined at the top and bottom of the numerical geometry to avoid spurious reflection effects and ensure the vanishing of the electric field. An air refractive index has been considered as unitary, while Ti permittivity was taken from ref (51). In modeling the porous titanium oxynitride NTs, the space-dependent 3D permittivity ε_NT_ introduced in the main text (see eq 1) was built, with TiN permittivity from ref (24) associated with the metallic phase, and air properties associated with voids. The content of O, alongside the small presence of TiO_2_ and Ti_2_O_3_ in the NT material, was neglected in defining the metal content permittivity upon the assumption that TiN gives the prominent contribution to the plasmonic character of the tubes. To numerically create the nanoporosity of the permittivity map, ε_NT_, a fictive stationary diffusion problem was set across the NT numerical domain, built as a regular tube-shaped geometry. This was done by means of the Transport of Diluted Species module embedded in COMSOL, to be solved upstream, before the electromagnetic study. The solver computes a concentration field *c* fulfilling the equation ∇*c* = 0, where a spatial gradient is created by imposing fixed concentrations at the outer and inner surfaces of the tube. These concentrations are defined, respectively, as a spatial random function, *R*(*x*,*y*,*z*), varying between 0 and 1 with uniform probability, and its complementary, 1 – *R*(*x*,*y*,*z*). These BCs enforce diffusion to occur also in the radial direction across the structure. A no-flux BC is imposed at the top and bottom surfaces of the NT. Solving for such a diffusion problem provides with a 3D random pattern ranging between 0 and 1 encoded in the concentration *c*, to be used to create the permittivity map for the electromagnetic study featuring deep subwavelength porosity. To tailor the degree of porosity given a threshold parameter value *ths*, the corresponding permittivity across the porous NT is defined according to eq 1 from the main text. Importantly, since such porosity of the permittivity is built from the numerical solution of the transport problem defined for variable c, the degree of porosity (i.e., the pore size) is directly controlled by the mesh of the model. A fine mesh was thus built across the nanoporous NT, with 40 edge elements along the perimeter of the tube transverse sections and a 20 nm maximum element size (comparable to the experimental pore size) along the longitudinal tube dimension.

Besides such an original approach, as mentioned in comments on Figure 4 from the main text, Effective Medium Theory (EMT) was employed to analyze the role of nanoporosity. The Bruggeman formalism was implemented,^46^ providing ε_NT_ as a homogeneous effective permittivity, written as a combination of the optical properties of the plasmonic phase (TiN) and those of air, weighted over the metal-to-void volume ratio *f* (corresponding therefore to a volume fraction of air of 1 – *f*). In formulas, ε_NT_ solves the following equation:^46^
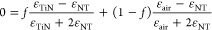


## Supporting Information Available

Detailed synthesis procedure, SEM and TEM images, dimensional parameters, and optical properties of TiO_2_ NTs; Additional details of nitridation procedure and lattice parameters of nitridated NTs; SEM and TEM images of NTs nitridated at 700 °C; Spectrum of the white LED; Additional details of photothermal experiments; Emissivity of nitridated NTs (PDF)
