# Supplementary material for: Nanoporous Titanium Oxynitride Nanotube Metamaterials with Deep Subwavelength Heat Dissipation for Perfect Solar Absorption
Source: ACS Photonics. 2023 Sep 8;10(9):3291–301. doi: 10.1021/acsphotonics.3c00731 (PMC10515634; doi:10.1021/acsphotonics.3c00731)
Supplement: Supplementary file 1 — ph3c00731_si_001.pdf [file ph3c00731_si_001.pdf]

Supporting Information

Nanoporous Titanium Oxynitride Nanotube Metamaterials with Deep Subwavelength Heat Dissipation for Perfect Solar Absorption

Morteza Afshar,[1,2] Andrea Schirato,[3,4,5] Luca Mascaretti,[1] S. M. Hossein Hejazi,[1,6] Mahdi Shahrezaei,[1,2] Giuseppe Della Valle,[3,7] Paolo Fornasiero,[8] Štěpán Kment,[1,6] Alessandro Alabastri,[5,] and Alberto Naldoni[1,9,]**

[1]Czech Advanced Technology and Research Institute, Regional Centre of Advanced Technologies and Materials Department, Palacký University Olomouc, Šlechtitelů 27, Olomouc 78371, Czech Republic

[2]Department of Physical Chemistry, Faculty of Science, Palacký University, 17. listopadu 1192/12, 779 00 Olomouc, Czech Republic

[3]Department of Physics, Politecnico di Milano, Piazza Leonardo da Vinci, 32, 20133 Milano, Italy

[4]Istituto Italiano di Tecnologia, via Morego 30, 16163, Genoa, Italy

[5]Department of Electrical and Computer Engineering, Rice University, 6100 Main Street, Houston, Texas 77005, United States

[6]CEET, Nanotechnology Centre, VŠB-Technical University of Ostrava, 17 Listopadu 2172/15, Ostrava-Poruba 708 00, Czech Republic

[7]Istituto di Fotonica e Nanotecnologie - Consiglio Nazionale delle Ricerche, Piazza Leonardo da Vinci, 32, I-20133 Milano, Italy

[8]Department of Chemical and Pharmaceutical Sciences, INSTM and ICCOM-CNR, University of Trieste, via L. Giorgieri 1, Trieste 34127, Italy

[9]Department of Chemistry and NIS Centre, University of Turin, Turin 10125, Italy

e-mail: alberto.naldoni@unito.it; alessandro.alabastri@rice.edu

This Supporting Information contains 9 pages, 8 figures and 2 tables.

Fabrication of TiO_2 nanotube arrays.

Highly spaced TiO_2 NTs with relatively ordered arrangement were produced by using different potentials of 20 (Figures S1a and S1b), 25 (Figures S1c and S1d), and 30 V (Figures S1e and S1f) applied for 6 h at 50 °C as well as 60 V applied for 6 h (Figures S1g–S1h) at 40 °C. The slightly higher anodization temperature employed at lower potentials (20, 25, and 30 V) ensured a good quality of the NT morphology eliminating "misfitting" tubes (Figure S2a), and the obtained samples anodized at 20, 25, 30, and 60 V are denoted as #1, #2, #3, and #4, respectively. The applied potential had a substantial impact on the growth of NTs and their diameter (Figure S1j). By increasing the anodization voltage, closely packed separated NTs with thinner walls (20 and 25 V) convert into completely separated NTs (30 and 60 V) with bigger wall thickness and intertube distances. In addition, the average diameter and length of NTs increased from ≈ 85 (Figures S1a and S1b) to ≈245 nm (Figures 1g and 1h) and from ≈1 to ≈5 μm, respectively, whereas further increments in voltage (≥70 V) resulted in discontinuous NTs embedded in a sponge-like layer (Figure S2b). TEM micrographs for TiO_2 NTs anodized at 60 V for 6 h were measured to study a single NT (Figure S1i). It is clear that such NT is made of a non-porous and uniform surface with outer and inner diameters of 275 and 245 nm at the top and 326 and 180 nm at the bottom, respectively.

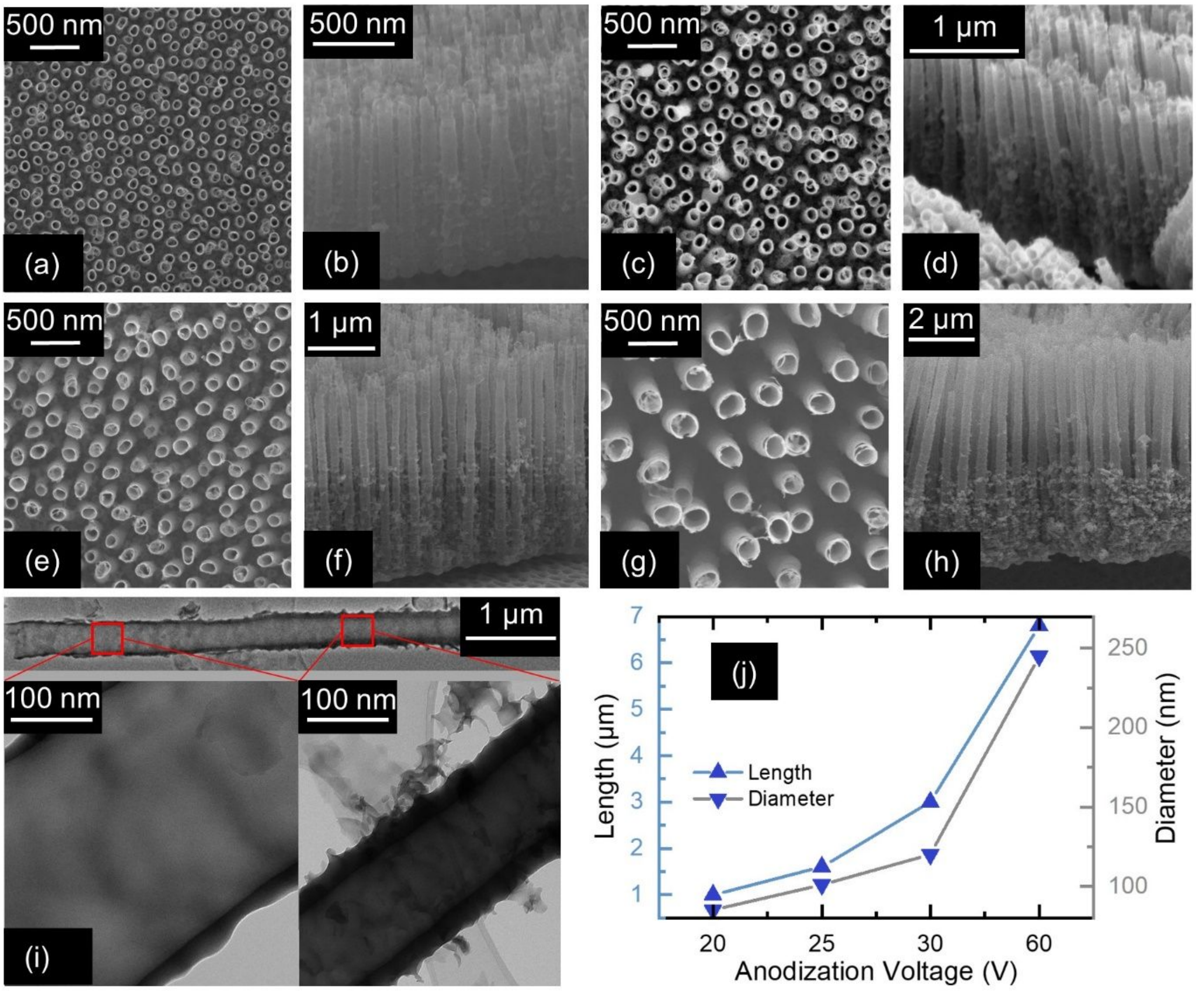

Figure S1. (a-h) SEM top views (first and third columns) and cross-sectional (second and forth columns) images of samples (a-f) anodized at 50 °C for 6 h at (a-b) 20 V, (c-d) 25 V, (e-f) 30 V, (g-h) anodized at 40 °C and 60 V and for 6 h. (i) TEM images of as-prepared TiO_2 nanotubes anodized at 60 V for 6h. (j) effects of the applied potential and anodization duration on the nanotubes average diameter and length.

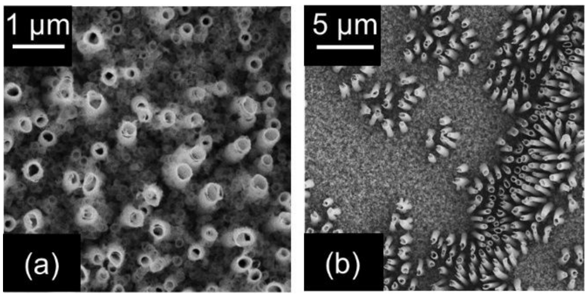

Figure S2. SEM images of (a) misfitting tubes anodized at 40 °C and 30 V (b) discontinuous nanotubes anodized at 40 °C and 70 V.

Optical absorption of as-anodized TiO_2 nanotubes.

Modifications to the morphology of NTs resulting from different anodization potentials can affect their optical properties (Figure S3). Overall, samples anodized at different voltages showed differing absorption spectra in the visible-to-NIR range. In addition, as-anodized TiO_2 NTs have diverse absorption spectra, making them have different colors, and the improved light-scattering and decreased light reflection characteristics of NT arrays leads to over 70% of light absorption up to 1100 nm which is very useful in photoconversion applications.[1] Specifically, sample anodized at 60 V exhibited gray with green tint color, and two narrow and broadband absorption peaks, respectively, in the UV range with an absorption edge at around 390 nm and the visible range. The former is related to the bandgap energy of TiO_2 which is 3.24 eV while the latter is relevant to the specific morphology of NTs.[2] On the other hand, the samples anodized at 20, 25 and, 30 V have metallic, yellow, and goldish colors, respectively, while their absorption peaks in the visible shifted toward shorter wavelengths and were monotonically decreased by reducing the anodization voltage. Light absorption is slightly enhanced at higher voltages because the increased voltage leads to NTs with a larger diameter and subsequently greater air volume inside and between the NTs, leading to a lower impedance mismatch and an increase in absorption. Although TiO_2 NT arrays demonstrated enhancement in light harvesting, however, their large band gap hinders the attainment of unitary absorption.

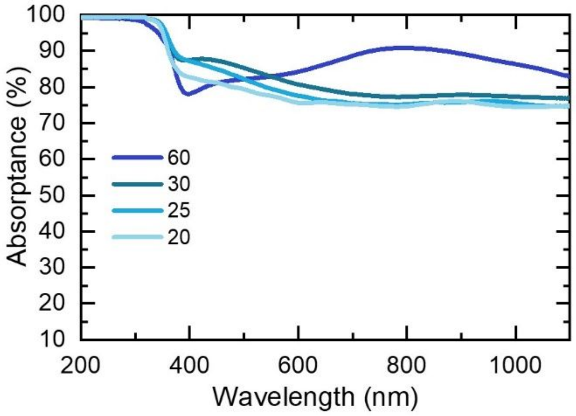

Figure S3. Absorption spectrum of as-anodized nanotube arrays anodized for 6 h at different voltages of 20, 25, 30, and 60 V.

Transformation of TiO_2 nanotubes to Titanium Oxynitride (TiO_xN_y) nanotube arrays.

One of the challenges in transforming TiO_2 NT to TiN NT arrays during the thermal treatment at high temperature under an NH_3 atmosphere is the structural decay and detachment of NTs from the substrate due to sintering effect or collapse.[3] To address this

issue, a preannealing process at a lower temperature was implemented to effectively improve the mechanical stability of TiO_2 NT arrays, allowing to preform successful nitridation process. Without air annealing, indeed, the NTs would peel off from the substrate after nitridation (not shown). Therefore, to prepare NT-based photothermal materials as solar absorbers, as-anodized TiO_2 NTs were treated by a two-step crystallization and reduction process. As-anodized NTs were first annealed in air at 450 °C to obtain the anatase crystal structure and to stabilize the NT array on the Ti substrate before further treatment. Subsequently, to convert TiO_2 into TiO_xN_y, NTs anodized at 60 V were nitridated under NH_3 at two different temperatures of 700 and 900 °C. On the other hand, NTs anodized at lower potentials were nitridated only at 700 °C, because nitridation at 900 °C led to collapse and peel off from the substrate. The obtained samples anodized at 20, 25, and 30 V and nitridated at 700 °C were labeled as #1, #2, and #3, respectively. Meanwhile, samples anodized at 60 V and subsequently nitridated at 700 and 900 °C were labeled as #4 and #5, respectively.

Lattice parameter. Bragg's law for the cubic crystal systems was employed to determine the lattice parameter of nitridated samples (Table S1), enabling a better differentiation between TiN and TiO due to their closely situated pick positions. The average lattice parameter extracted from the XRD pattern for the sample nitridated at 700 °C for the (111) and (200) reflections was ~ 4.18 Å which is in close accordance with that of TiO, α_{TiO}= 4.1770 (JCPDS card no. 00-08-0117). Moreover, considering the presence of TiO_2 and Ti_2O_3, it, therefore, can be inferred that the sample nitridated at 700 °C has a higher level of oxygen with a trace amount of nitrogen. On the other hand, upon nitridation at a higher temperature (900 °C), the diffraction peaks of TiO_2 and Ti_2O_3 completely disappeared, while the Ti(O,N) peaks in comparison to the sample nitridated at 700 °C slightly shifted toward lower 2θ values (Figure 2b), indicating that the lattice parameter of the sample nitridated at 900 °C increased (~ 4.23 Å). In other words, by nitridation at the higher temperature, the amount of oxygen atom which has a smaller diameter decreased in the product whereas the amount of nitrogen atom which has a larger diameter increased, and consequently, the diffraction peak shifted toward larger values and closer to the standard position or lattice parameter of TiN, α_{TiN}= 4.2260 Å (JCPDS file no. 04-002-0575).

Table S1. Lattice parameter (α) of nitridated samples determined by Bragg's law for the cubic crystal systems.

Nitridation temperature (°C)	Miller indices	2θ (degree)	α (Å)	Average α (Å)
700	(111)	43.62	4.1721	4.1770
700	(200)	50.68	4.1820	
900	(111)	43.05	4.2242	4.2252
900	(200)	50.11	4.2262	

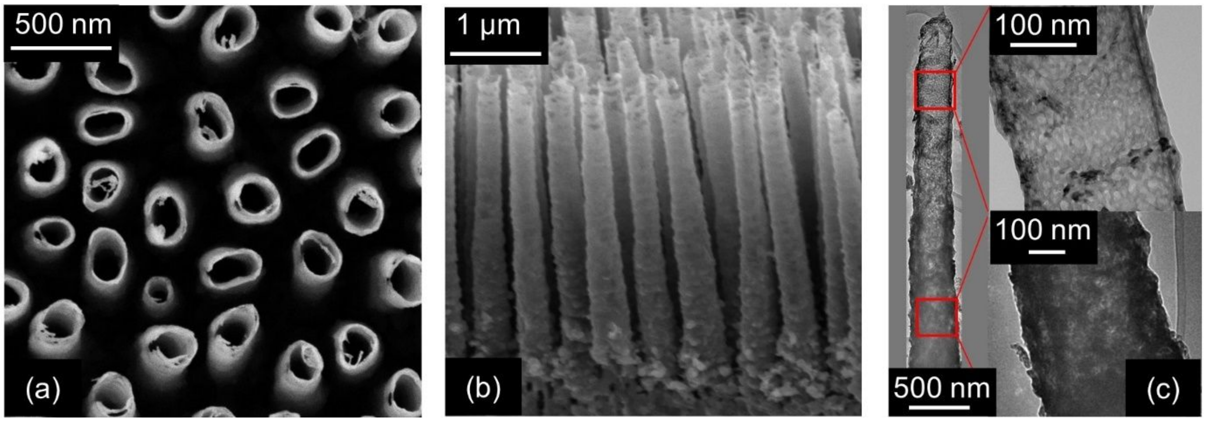

Figure S4. (a-b) SEM images (top (a) and cross-sectional (b) view) and (c) TEM micrographs of nanotubes anodized at 60 V for 6 h and nitridated at 700 °C.

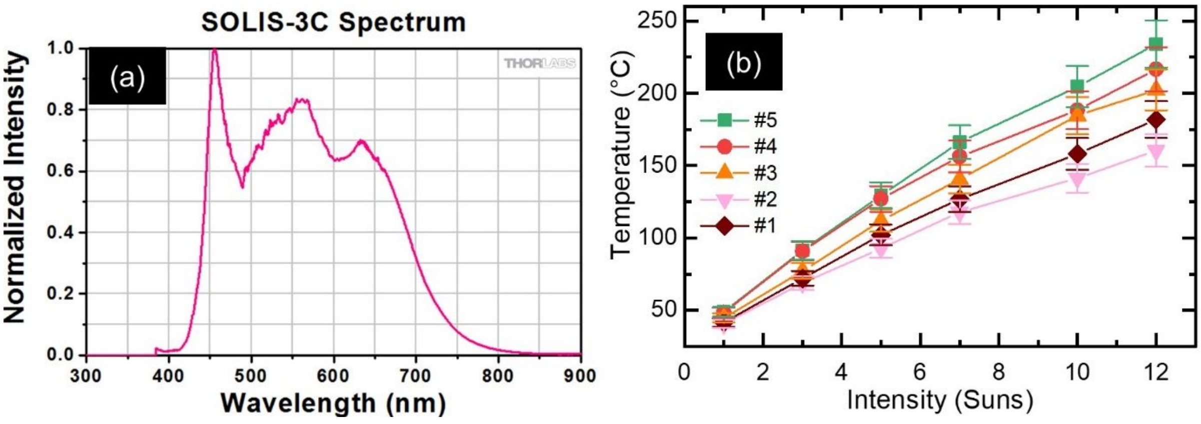

Figure S5. (a) The spectral characteristics of the white LED used for the photothermal measurement of nanotube arrays[4] and (b) the maximum temperature (T_{max}) attained by different samples under various intensities after 60 s of irradiation.

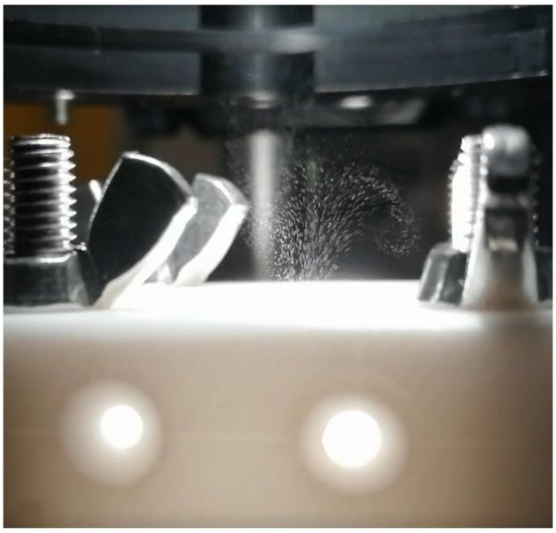

Figure S6. Photograph of the steam flow generated under 14 Suns illumination of the solar simulator with #5 nanotube arrays as a photothermal material.

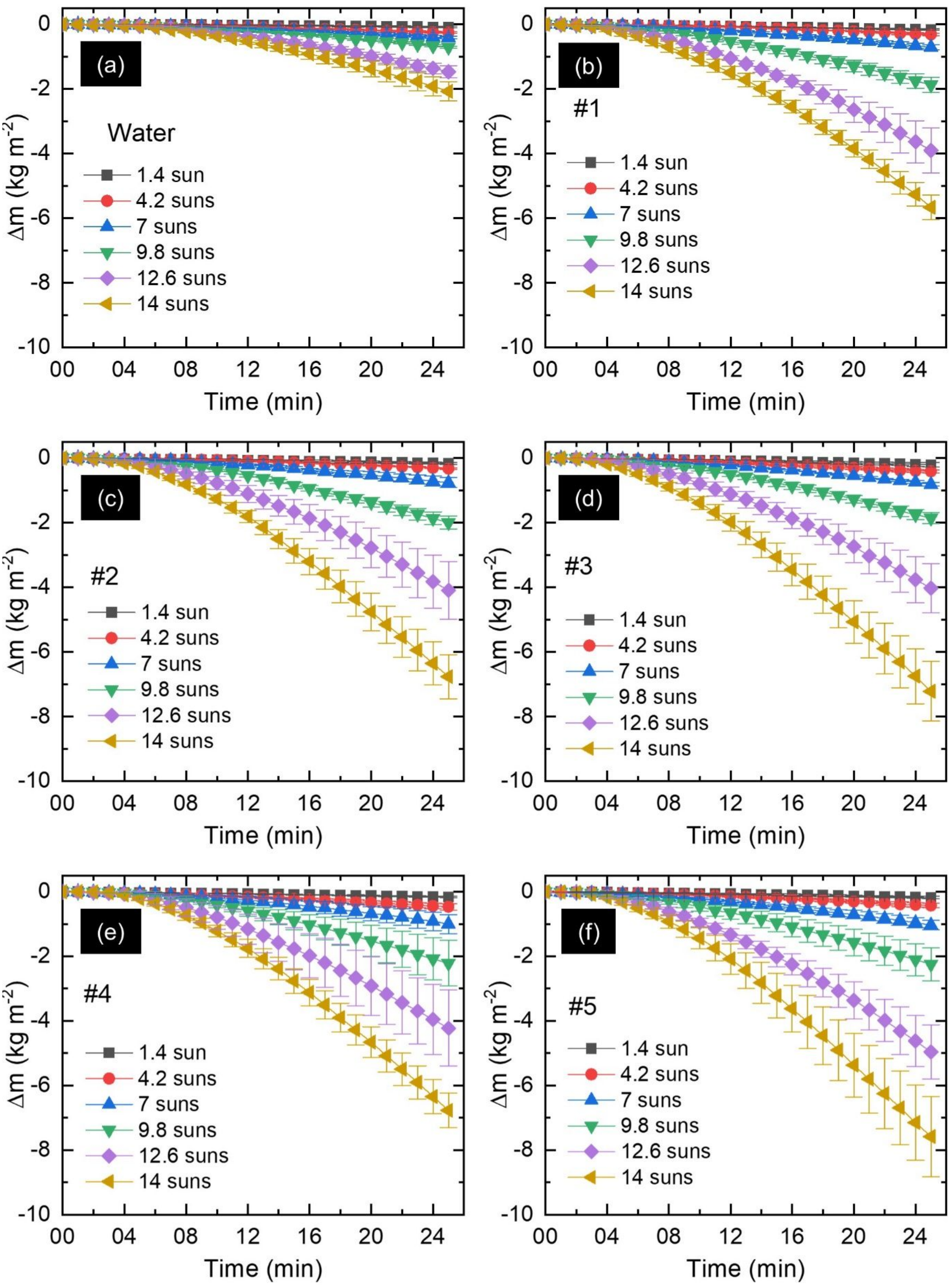

Figure S7. The time-dependent weight change of water under different irradiation intensities for (a) water–only and TiO_xN_y nanotube arrays of (b) #1, (C) #2, (d) #3 (e) #4, and (f) #5.

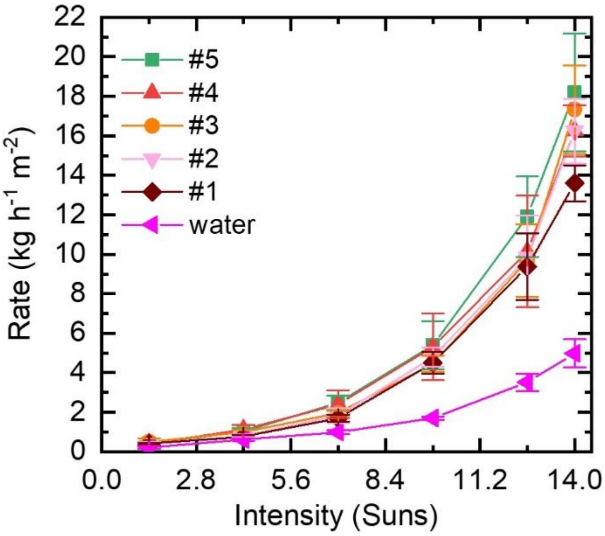

Figure S8. Comparison of water evaporation rate among different TiO_xN_y nanotube arrays.

Table S2. The emissivity of different TiO_xN_y nanotube arrays over the range of 8-14 µm.

Sample	Average emissivity
#1	0.314
#2	0.538
#3	0.834
#4	0.949
#5	0.972

Author Contributions

M. A. and A. S. contributed equally to this paper.

References

(1) Varghese, O. K.; Paulose, M.; Grimes, C. A. Long Vertically Aligned Titania Nanotubes on Transparent Conducting Oxide for Highly Efficient Solar Cells. *Nat. Nanotechnol.* **2009**, *4* (9), 592–597. https://doi.org/10.1038/nnano.2009.226.

(2) Tesler, A. B.; Altomare, M.; Schmuki, P. Morphology and Optical Properties of Highly Ordered TiO_2 Nanotubes Grown in NH_4F/*o*-H_3PO_4 Electrolytes in View of Light-Harvesting and Catalytic Applications. *ACS Appl. Nano Mater.* **2020**, *3* (11), 10646–10658. https://doi.org/10.1021/acsanm.0c01859.

(3) Al-Thabaiti, S. A.; Hahn, R.; Liu, N.; Kirchgeorg, R.; So, S.; Schmuki, P.; Basahel, S. N.; Bawaked, S. M. NH_3 Treatment of TiO_2 Nanotubes: From N-Doping to Semimetallic Conductivity. *Chem. Commun.* **2014**, *50* (59), 7960. https://doi.org/10.1039/c4cc02069c.

(4) https://www.thorlabs.com/thorproduct.cfm?partnumber=SOLIS-3C
